# Supplementary material for: Highly Effective Thermally Activated Delayed Fluorescence Emitters Based on Symmetry and Asymmetry Nicotinonitrile Derivatives
Source: Molecules. 2022 Nov 27;27(23):8274. doi: 10.3390/molecules27238274 (PMC9738715; doi:10.3390/molecules27238274)
Supplement: Supplementary file 1 [file molecules-27-08274-s001.zip › molecules-2039759-supplementary.pdf]

## Supplementary Materials

# Highly Effective Thermally Activated Delayed Fluorescence Emitters Based on Symmetry and Asymmetry Nicotinonitrile Derivatives

**Min Gyeong Choi [1], Chan Hee Lee [2,3], Chihaya Adachi [2,3] and Sae Youn Lee [1,*]**

[1] Department of Energy and Materials Engineering, Dongguk University, Seoul 04620, Republic of Korea
[2] Center for Organic Photonics and Electronics Research (OPERA), Kyushu University, 744 Motooka, Nishi-ku, Fukuoka 819-0395, Japan
[3] Department of Chemistry and Biochemistry, Kyushu University, 744 Motooka, Nishi-ku, Fukuoka 819-0395, Japan
* Correspondence: saeyounlee@dongguk.edu

Contents

1. General methods
2. Materials and synthesis
3. $^{1}$H NMR spectra
4. TD-DFT calculation data
5. Thermal and photophysical properties
6. Determination of rate constants
7. References

## 1. General methods

$^1$H NMR spectra were recorded on an Avance III 500 spectrometer (Bruker). Chemical shifts of $^1$H NMR signals were quoted to tetramethylsilane ($\delta$ = 0.00) as an internal standard. Matrix-assisted laser desorption ionization time-of-flight (MALDI-TOF) mass spectra were collected on IDSys (ASTA, Korea) using dithranol as the matrix. The UV/Vis absorption and PL spectra of organic films were measured with a UV-2550 (Shimadzu) and a FluoroMax-4 spectrofluorometer (Horiba Scientific), respectively. Luminescence intensity and lifetime of organic films were measured with a Streak camera (Hamamatsu Photonics C4334). The organic films were excited by an $N_2$ gas laser ($\lambda$ = 337 nm, pulse width = 500 ps, repetition rate 20 Hz) under a vacuum of < $4\times10^{-1}$ Pa. Samples were cooled down at 5 K with a cryostat (Iwatani Industrial Gases). The time-dependent density functional theory (TD-DFT) computations were performed on the Gaussian 09 program, using the B3LYP functional with the 6-31G(d) basis set [1,2].

## 2. Materials and synthesis

All reagents and solvents were purchased from Sigma-Aldrich, Tokyo Chemical Industry (TCI), or Alfa Aesar. The synthetic routes to obtain **ICzCN** and **ICzCYP** are outlined in Scheme S1. All reactions were performed under $N_2$ atmospheres in dry solvents. The final products were fully characterized by $^1$H NMR spectroscopy, MALDI-TOF mass spectrometry, and elemental analysis, as described below.

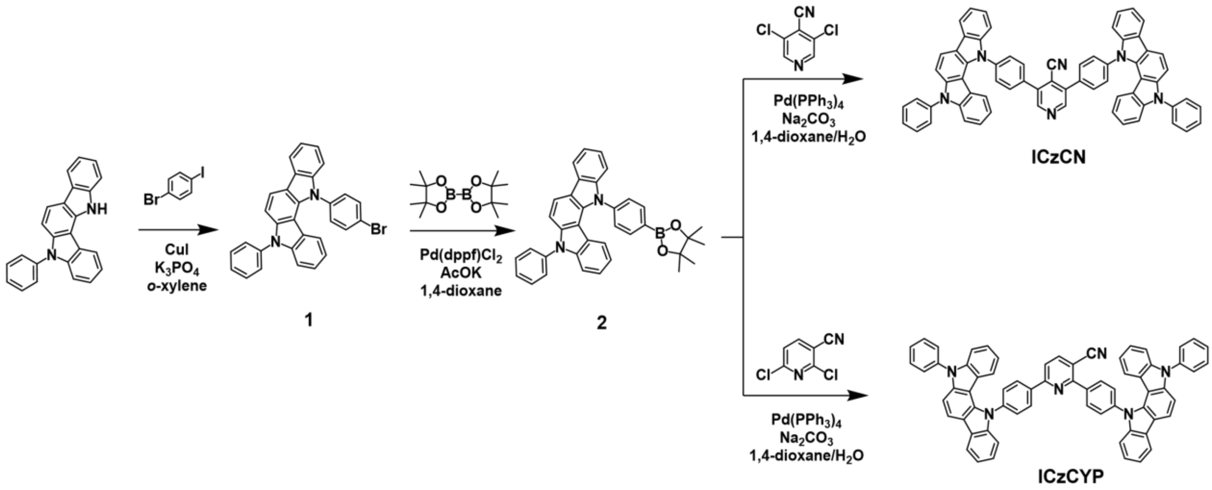


**Scheme S1.** Synthesis of **ICzCN** and **ICzCYP**.

**Synthesis of 12-(4-bromophenyl)-5-phenyl-5,12-dihydroindolo[3,2-*a*]carbazole (1)** [3].

5-phenyl-5,12-dihydroindolo[3,2-*a*]carbazole (8.98 g, 27 mmol), 1-bromo-4-iodobenzene (8.40 g, 29.7 mmol), copper iodide (0.37 g, 2.7 mmol) and potassium tribasic phosphate (5.73 g, 27 mmol) were dissolved in *o*-xylene (90 mL) in nitrogen atmosphere. To this solution, (±)-*trans*-1,2-diaminocyclohexane (0.31 mL, 2.7 mmol) was added and the mixture was refluxed for 6 h . After hot filtration with toluene, the reaction mixture was added into water, and then extracted with chloroform, and the combined organic layers were washed with water and dried over anhydrous $MgSO_4$. After filtration with Celite 545 and solvent removal, the crude material was purified with column chromatography on silica gel (eluent : dichloromethane/hexane, 1:3 *v*/*v*) and dried in a vacuum oven to obtain a white powder (yield = 3.95 g, 30%). $^1$H NMR (500 MHz, DMSO-$d_6$) data are as follows : $\delta$ 8.33 (dd, $J$ = 8.5, 3.0 Hz, 1H), 8.25 (d, $J$ = 7.5 Hz, 1H), 8.10 (d, $J$ = 8.5 Hz, 1H), 7.94 (d, $J$ = 8.5 Hz, 1H), 7.74 (t, $J$ = 8.0 Hz, 2H), 7.67-7.61 (m, 4H), 7.51 (d, $J$ = 8.5 Hz, 1H), 7.38-7.27 (m, 6H), 6.86 (dd, $J$ = 15.0, 8.5 Hz, 1H), 6.01 (t, $J$ = 8.5 Hz, 1H).

**Synthesis of 5-phenyl-12-(4-(4,4,5,5-tetramethyl-1,3,2-dioxaborolan-2-yl)phenyl)-**

**5,12-dihydroindolo[3,2-*a*]carbazole (2)** [4].

**1** (3.95 g, 8.1 mmol), bis(pinacolato)diboron (2.26 g, 12.2 mmol), potassium acetate (2.38 g, 24.3 mmol) and 1,1′-bis(diphenylphosphino)ferrocene]dichloropalladium(II) (0.41 g, 0.6 mmol) was dissolved in 1,4-dioxane (79 mL) in nitrogen atmosphere. The reaction mixture was stirred and refluxed for 24 h. After cooling to room temperature, the reaction mixture was then poured into water. The product was extracted with chloroform, and the combined organic layers were washed with water and dried over anhydrous $MgSO_4$. After filtration with Celite 545 and solvent removal, the crude material was purified with column chromatography on silica gel (eluent : ethyl acetate/hexane, 1:10 *v*/*v*) and dried in a vacuum oven to obtain a white powder (yield = 2.60 g, 60%). $^1$H NMR (500 MHz, DMSO-$d_6$) data are as follows : $\delta$ 8.33 (d, $J$ = 8.5 Hz, 1H), 8.25 (d, $J$ = 7.5 Hz, 1H), 8.01 (d, $J$ = 8.0 Hz, 2H), 7.73 (t, $J$ = 7.5 Hz, 2H), 7.69 (d, $J$ = 8.0 Hz, 2H), 7.66 (d, $J$ = 7.5 Hz, 2H), 7.62 (t, $J$ = 7.5 Hz, 1H), 7.38-7.24(m, 6H), 6.74-6.69 (m, 1H), 5.89 (d, $J$ = 8.5 Hz, 1H), 1.39 (s, 12H).

**Synthesis of 3,5-bis(4-(5-phenylindolo[3,2-*a*]carbazol-12(5*H*)-yl)phenyl)isonicotinonitrile (ICzCN)**.

3,5-dichloro-4-pyridinecarbonitrile (0.29 g, 1.7 mmol), **2** (1.91 g, 3.6 mmol), tetrakis(tri-phenylphosphine)palladium(0) (0.20 g, 0.2 mmol) were dissolved in dry 1,4-dioxane (29 mL). To the solution were added an aqueous sodium carbonate (2 M, 15 mL). The reaction mixture was stirred for 24 h 90°C. After cooling to room temperature, the reaction mixture was then poured into water. The product was extracted with chloroform, and the combined organic layers were washed with water and dried over anhydrous $MgSO_4$. After filtration with Celite 545 and solvent removal, the crude material was purified with column chromatography on silica gel (eluent : dichloromethane/hexane, 6:1 *v*/*v*) and dried in a vacuum oven to obtain a yellow powder (yield = 1.08 g, 69%). This compound was further purified by temperature-

gradient sublimation in vacuum. $^1$H NMR (500 MHz, DMSO-$d_6$) data are as follows : δ 9.17 (s, 2H), 8.37 (d, *J* = 8.5 Hz, 2H), 8.30 (d, *J* = 7.5 Hz, 2H), 8.19 (d, *J* = 8.5 Hz, 4H), 7.99 (d, *J* = 8.5 Hz, 4H), 7.74 (t, *J* = 8.0 Hz, 4H), 7.68 (d, *J* = 7.5 Hz, 4H), 7.62 (t, *J* = 7.5 Hz, 2H), 7.46-7.40 (m, 4H), 7.39 (t, *J* = 7.5 Hz, 2H), 7.32 (d, *J* = 9.0 Hz, 2H), 7.26 (t, *J* = 6.0 Hz, 4H), 6.96 (t, *J* = 8.0, 2.0 Hz, 2H), 6.13 (d, *J* = 8.0 Hz, 2H). MS : *m/z* 916.97 [M]$^+$; calcd. 916.33.

**Synthesis of 2,6-bis(4-(5-phenylindolo[3,2-*a*]carbazol-12(5*H*)-yl)phenyl)nicotino-nitrile (ICzCYP).**

This compound was synthesized by following a procedure similar to that employed for the synthesis of **ICzCN**, except that 2,6-dichloro-6-pyridinecarbonitrile (0.43 g, 2.5 mmol) was used instead of 3,5-dichloro-4-pyridinecarbonitrile. The crude material was dried in a vacuum oven to obtain a yellow powder (yield = 1.92 g, 96%). This compound was further purified by temperature-gradient sublimation in vacuum. $^1$H NMR (500 MHz, $CDCl_3$) data are as follows : δ 8.54 (d, *J* = 8.0 Hz, 2H), 8.41(d, *J* = 8.0 Hz, 2H), 8.33 (d, *J* = 8.0 Hz, 1H), 8.19 (m, 4H), 8.07 (d, *J* = 8.0 Hz, 1H), 7.89 (d, *J* = 8.0 Hz, 2H), 7.85 (d, *J* = 8.0 Hz, 2H), 7.65 (t, *J* = 8.0 Hz, 4H), 7.61-7.22 (m, 22 H), 6.94 (t, *J* = 8.0 Hz, 1H), 6.80 (t, *J* = 8.0 Hz, 1H), 6.26 (d, *J* = 8.0 Hz, 2H). MS : *m/z* 916.76 [M]$^+$; calcd. 916.33.

## 3. $^1$H NMR spectra

**(a)**

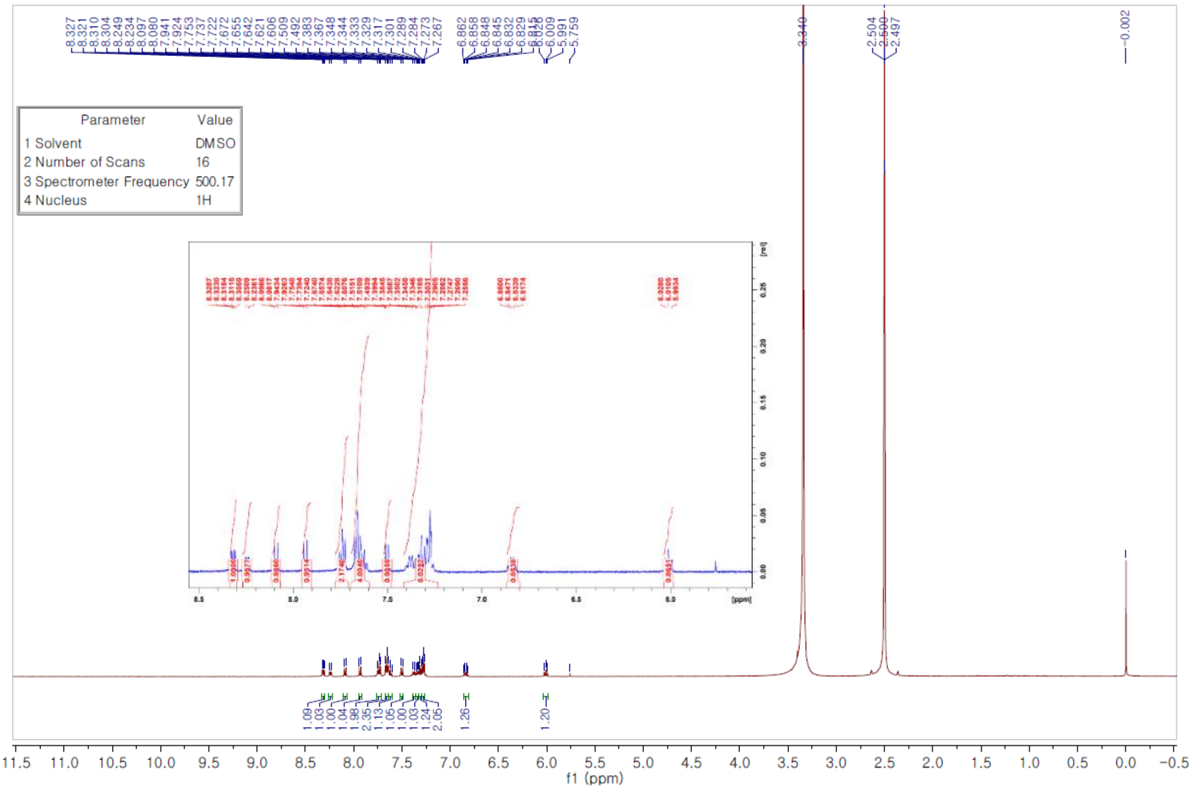


**(b)**

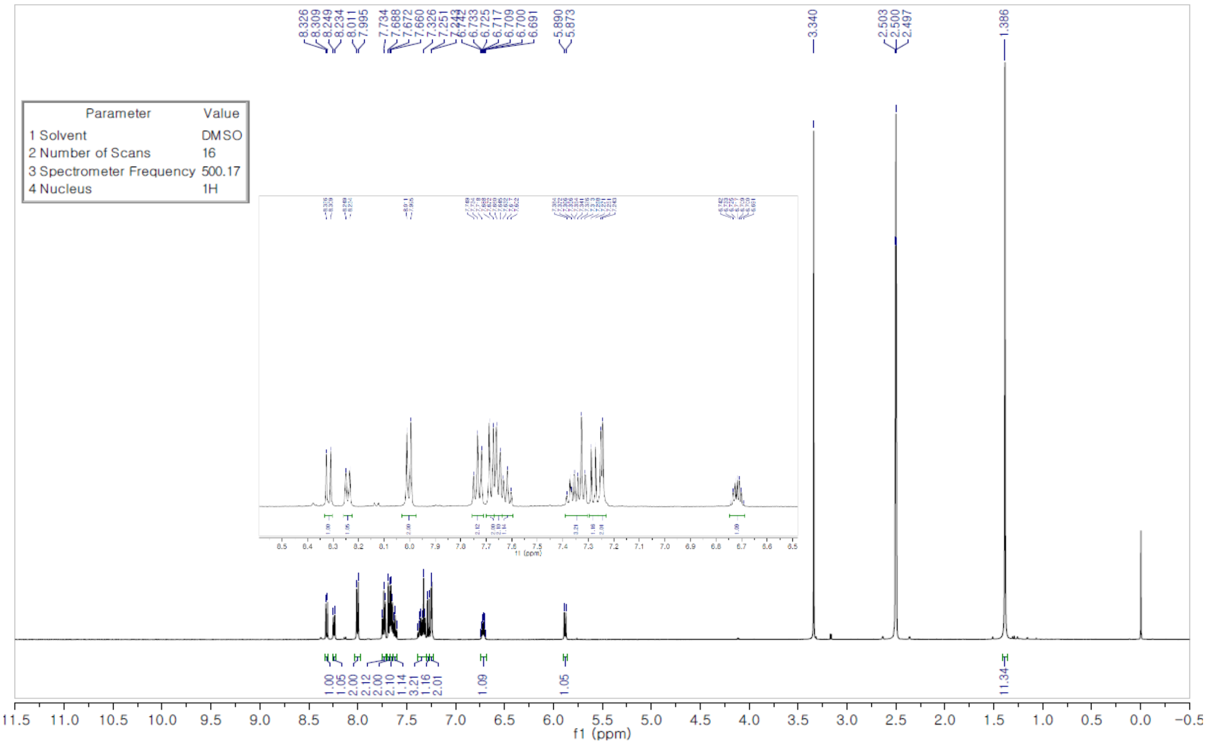

**(c)**

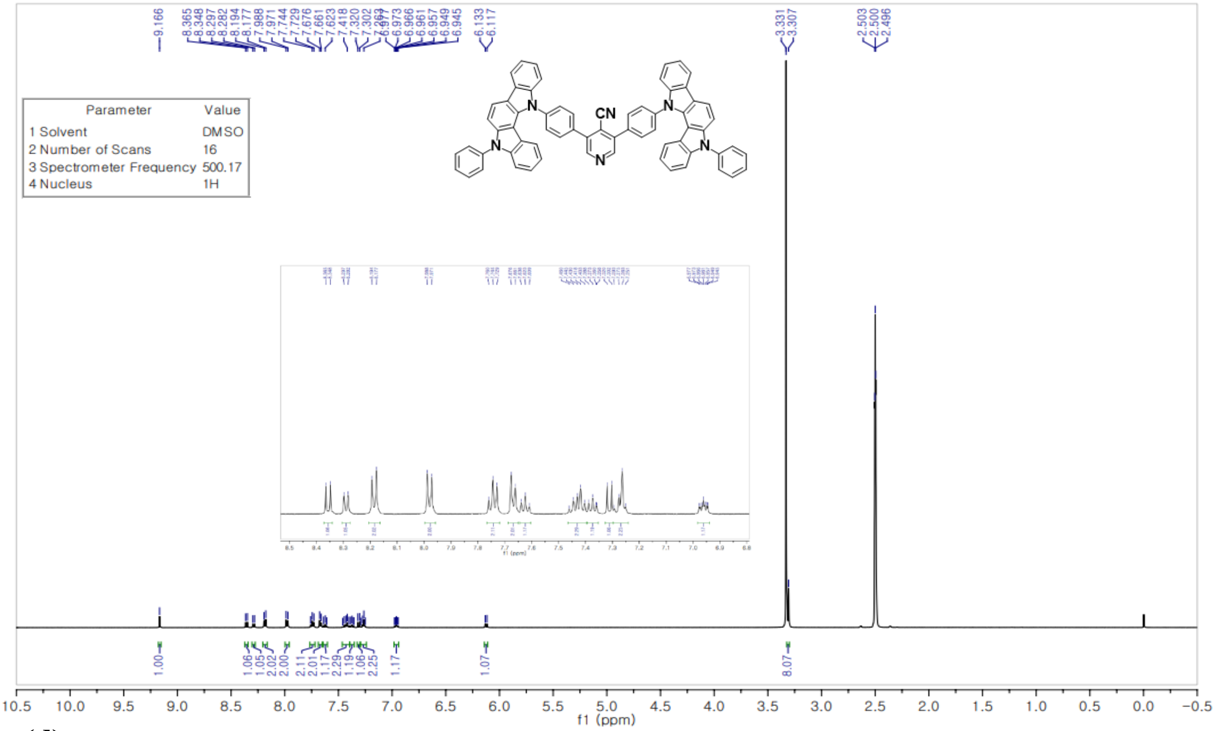


**(d)**

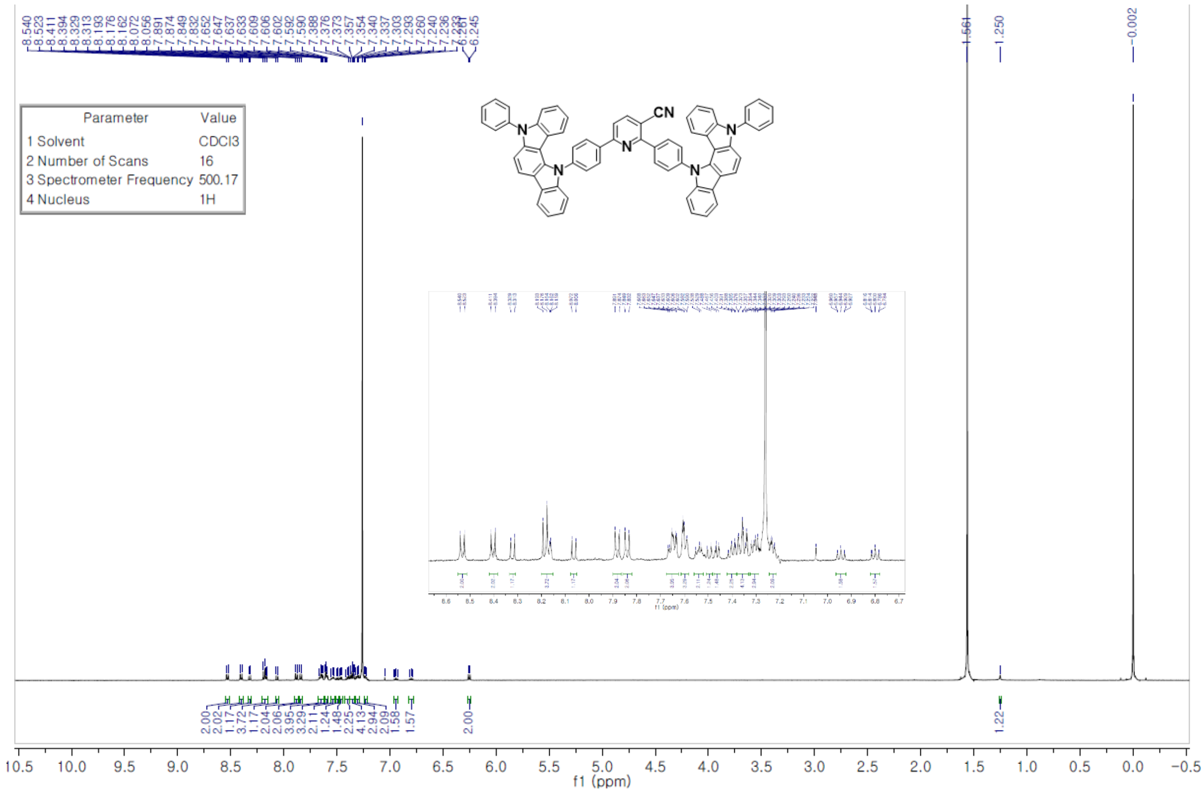


**Figure S1.** $^1$H NMR of (a) **1,** (b) **2,** (c) **ICzCN**, and (d) **ICzCYP**.

## 4. TD-DFT calculations data

**Table S1.** Triplet and singlet excitation energies (vertical transition), oscillator strength (*f*), and transition configurations of the nicotinonitrile derivatives **ICzCN** and **ICzCYP** calculated by TD-DFT at the B3LYP/6-31G(d).

| compound | state | E [eV] | f | Main configuration [a] | | $\Delta E_{ST}$ [eV] |
|---|---|---|---|---|---|---|
| **ICzCN** | $S_1$ | 2.503 | 0.0043 | HOMO → LUMO | 0.7050 | 0.008 |
| | $S_2$ | 2.588 | 0.0105 | HOMO −1→ LUMO | 0.7036 | |
| | $T_1$ | 2.495 | 0.0000 | HOMO → LUMO | 0.6999 | |
| | $T_2$ | 2.568 | 0.0000 | HOMO −1→ LUMO | 0.6798 | |
| **ICzCYP** | $S_1$ | 2.484 | 0.0076 | HOMO → LUMO | 0.7028 | 0.027 |
| | $S_2$ | 2.590 | 0.0274 | HOMO −1→ LUMO | 0.7012 | |
| | $T_1$ | 2.457 | 0.0000 | HOMO → LUMO | 0.6074 | |
| | $T_2$ | 2.503 | 0.0000 | HOMO −1→ LUMO | 0.4622 | |

[a] HOMO → LUMO represents the HOMO to LUMO transition. Excitation configurations with the highest contributions are presented, together with the corresponding transition symmetry and nature of the involved orbitals.

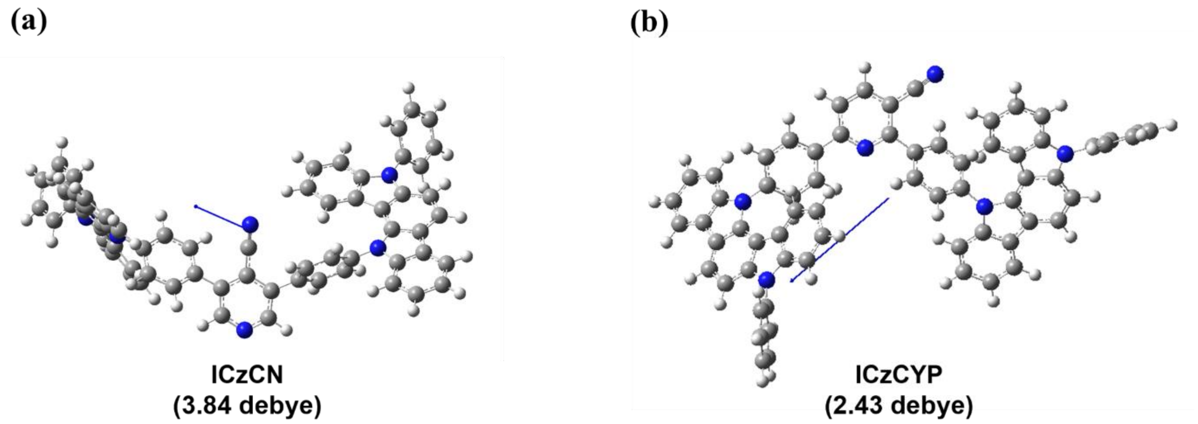


**Figure S2.** The transition dipole moments of (a) **ICzCN** and (b) **ICzCYP** in optimized molecular structure.

## 5. Thermal and photophysical properties

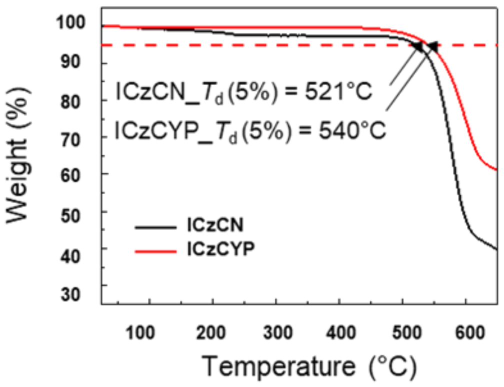


**Figure S3.** TGA profiles of **ICzCN** (black) and **ICzCYP** (red).

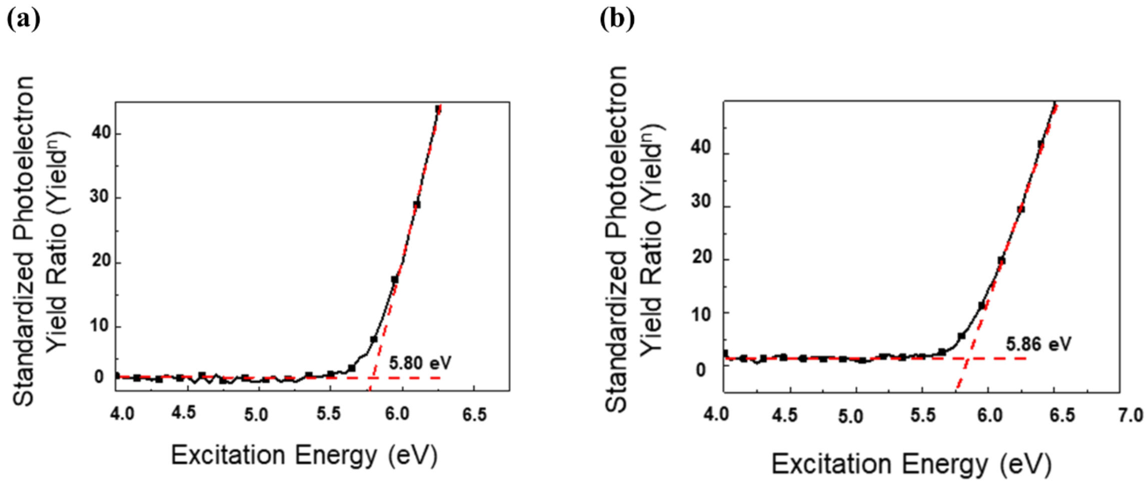


**Figure S4.** Photoelectron yield spectra of (a) **ICzCN** and (b) **ICzCYP**.

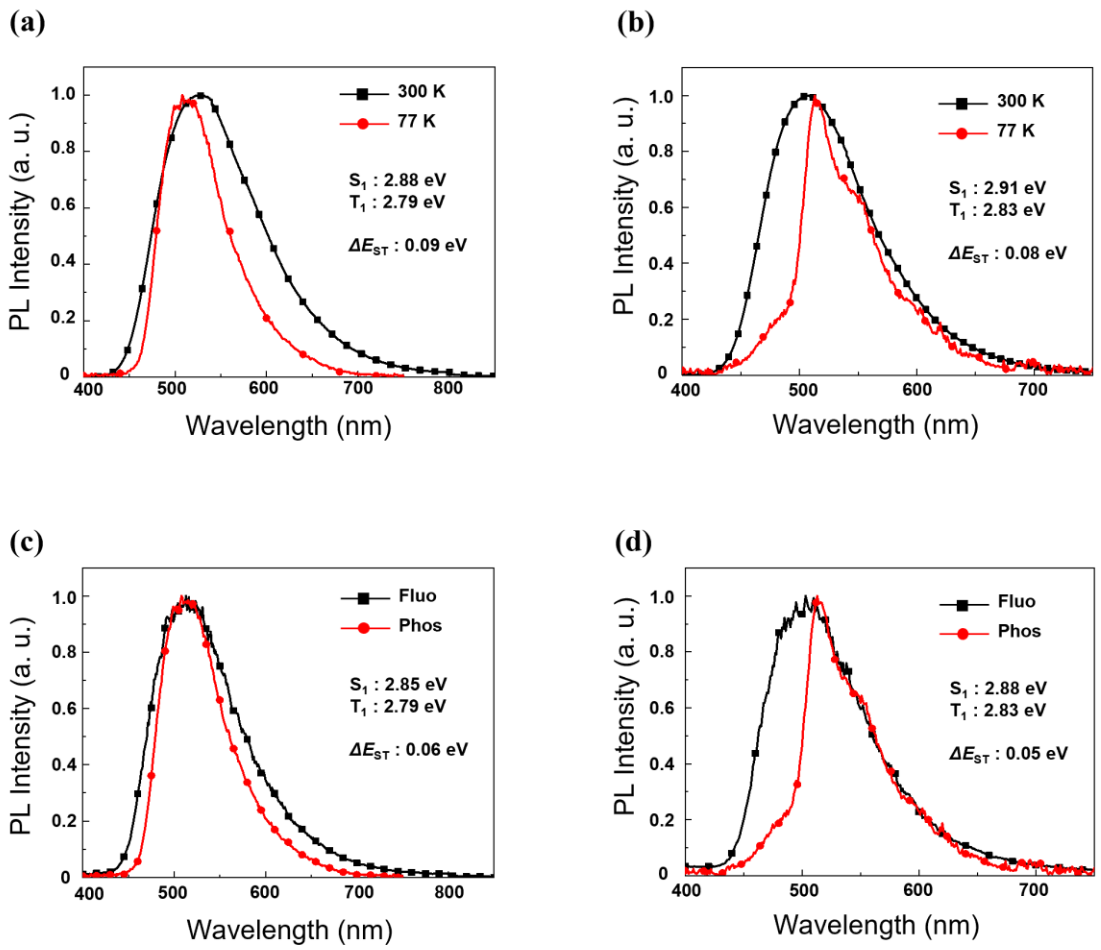


**Figure S5.** PL spectra of prompt fluorescence at 300 K (black), phosphorescence at 77 K (red) for (a) **ICzCN** and (b) **ICzCYP** and fluorescence (black) and phosphorescence (red) at 77 K for (c) **ICzCN** and (d) **ICzCYP**.

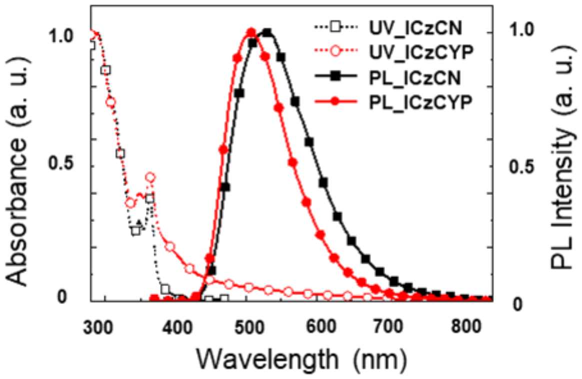


**Figure S6.** UV-Vis and PL spectra of (a) **ICzCN** and (b) **ICzCYP** neat films.

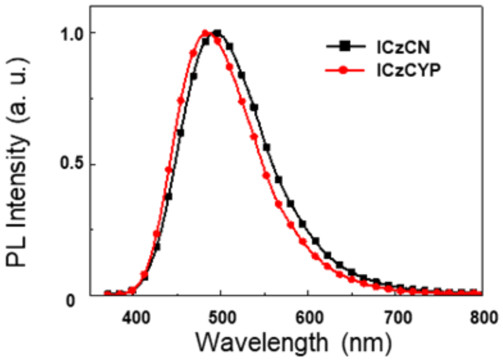


**Figure S7.** PL spectra of 12 wt% (a) **ICzCN** and (b) **ICzCYP** doped films in PPF host.

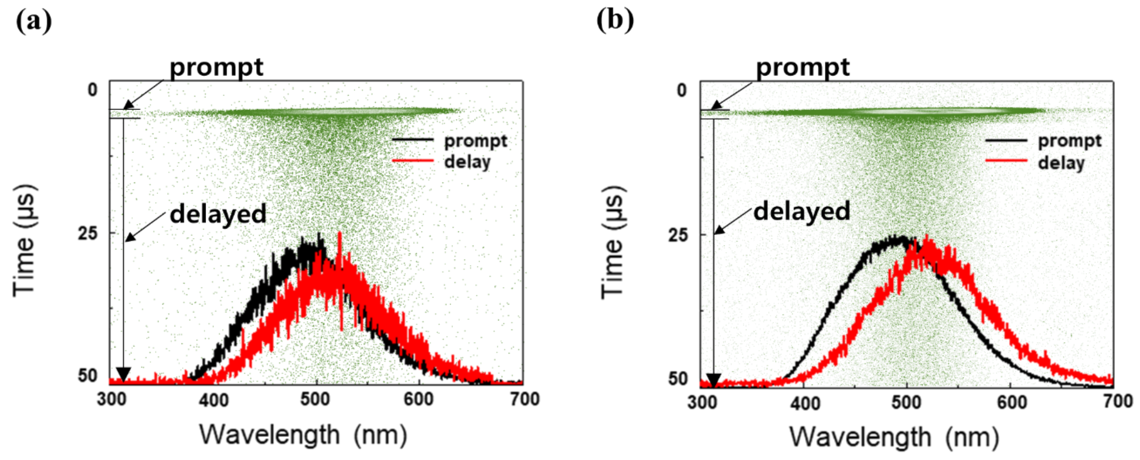


**Figure S8.** Streak images and time-resolved PL spectra of 12 wt% (a) **ICzCN**:PPF and (b) **ICzCYP**:PPF co-deposited films.

**Table S2.** Photophysical properties of **ICzCN** and **ICzCYP** in solution[a)], neat film and 12 wt% doped film in PPF host.

| compound | $\lambda_{abs}$ [nm] | | $\lambda_{PL}$ [nm] | | $\Phi_{PL}$ [%] | | |
|---|---|---|---|---|---|---|---|
| | solution | neat | solution | neat | solution | | 12 wt% doped film[b)] |
| | | | | | air | $N_2$ | $N_2$ |
| **ICzCN** | 371 | 379 | 489 | 529 | 18 | 63 | 76 |
| **ICzCYP** | 370 | 376 | 475 | 508 | 23 | 52 | 58 |

[a)] in toluene, $10^{-4}$ M

[b)] in PPF host

## 6. Rate constants

Rate constants of prompt and delayed PL components were determined as follows.

1) Radiative decay rate constant of fluorescence

$$k_r^S = \Phi_p/\tau_p \quad (1)$$

2) Radiative decay rate constant of delayed fluorescence

$$k_d = 1/\tau_d \quad (2)$$

3) Nonradiative decay rate constant of $T_1$ state

$$k_{nr,T} = k_d - (\Phi_p \times k_{RISC}) \quad (3)$$

4) Rate constant of ISC

$$k_{ISC} = k_p - k_r^S - k_{nr}^S,\ k_{nr}^S \approx 0 \quad (4)$$

5) Rate constant of RISC

$$k_{RISC} = (k_p \times k_d \times \Phi_d)/(k_{ISC} \times \Phi_p) \quad (5)$$

6) Quantum efficiency of ISC from $S_1$ to $T_1$ state

$$\Phi_{ISC} = 1 - \Phi_p \quad (6)$$

7) Quantum efficiency of RISC from $T_1$ to $S_1$ state

$$\Phi_{RISC} = \Phi_d/\Phi_{ISC} \quad (7)$$

## 7. References


1. Lee, C.H.; Choi, S.H.; Oh, S.J.; Lee, J.H.; Shim, J.W.; Adachi, C.; Lee, S.Y. Highly effective organic light-emitting diodes containing thermally activated delayed fluorescence emitters with horizontal molecular orientation. *RSC Adv.* **2020**, 10, 42897-42902.

2. Choi, S.H.; Lee, C.H.; Adachi, C.; Lee, S.Y. Molecular design of highly effective thermally activated delayed fluorescence emitters based on *ortho*-substituted donor-acceptor-donor pyridinecarbonitrile derivatives and their application for high-performance OLEDs. *Dyes and Pigments* **2019**, 171, 107775.

3. Liang, X.; Wang, Z.; Wang, L.; Hanif, M.; Hu, D.; Su, S.; Xie, Z.; Gao, Y.; Yang, B.; Ma, Y. Tailoring Excited State Properties and Energy Levels Arrangement via Subtle Structural Design on D- π -A Materials. *Chin. J. Chem.* **2017**, 35, 1559-1568.

4. Miralles, N.; Romero, R.M.; Fernández, E.; Muñiz, K. A mild carbon-boron bond formation from diaryliodonium salts. *Chem. Commun.* **2015**, 51, 14068-14071.
